# Commentary: The influence of linear and nonlinear pedagogy on motor skill performance: the moderating role of adaptability

**DOI:** 10.3389/fpsyg.2026.1803932

**Published:** 2026-04-24

**Authors:** Gibson Moreira Praça, Yibeltal Abebe, Guilherme De Oliveira Santos Silva, Lucas Savassi Figueiredo, Sara Diniz, Layla Maria Campos Aburachid

**Affiliations:** 1 Department of Sports, Universidade Federal de Minas Gerais, Belo Horizonte, Brazil; 2 Department of Physical Education, Universidade Federal de Mato Grosso, Cuiabá, Brazil

**Keywords:** instruction, non-linear, pedagogy, soccer (football), sports, team sports

We read with great interest the article by Yang et al. (2025) “The influence of linear and nonlinear pedagogy on motor skill performance: the moderating role of adaptability,” published in Frontiers in Psychology. The study addresses an important and timely topic in motor learning and coaching science, offering a well-structured investigation grounded in ecological dynamics and supported by clear statistical analyses. It is also important to highlight the analysis of adaptability within the learning domain, as it addresses a key limitation of traditional dependent variables in intervention studies, which often fail to capture this fundamental characteristic of team sports.

However, after carefully examining both the main text and Supplementary material, we would like to raise several methodological and conceptual issues that may influence the interpretation of the results. Our intention is not to detract from the study's contribution but rather to promote methodological rigor and theoretical coherence in future research.

## 1 Possible theoretical inconsistency in the representation of linear pedagogy

The first concern relates to the conceptualization of the Linear Pedagogy (LP) condition. The study characterizes LP as a reductionist pedagogical approach that emphasizes “skill decomposition and repetitive part-task practice” (Yang et al., 2025, p. 2). Skill decomposition and part-task time indeed are traditional approaches within sports teaching contexts, for example, the direct instruction model (Metzler, 2011). Within these approaches, teachers typically demonstrate the target movement, skill, or concept, and then structure practice into organized time segments in which learners repeatedly perform the task while receiving frequent augmented feedback and encouragement (Metzler, 2011). Therefore, linear pedagogy still requires coaches to structure tasks and lessons to optimize learning; this process typically unfolds in a sequential manner over time, with a progressive increase in task difficulty and complexity until the final game is reached. However, as implemented (e.g., “dribbling around cones” or “fixed passing patterns,” as indicated in the supplementary material), the LP program may not fully represent the breadth of established linear pedagogical approaches.

To solve this issue, LP interventions should clearly include contextualized practice, feedback provision, and progressive tactical integration, as traditionally assumed by models such as direct instruction (Metzler, 2011). Without this, the interpretation of LP intervention outcomes becomes problematic because it represents a narrow and possibly limited operationalization of linear pedagogy. While teacher-directed, structured, and repetitive instructional approaches are well-established within physical education and skill acquisition traditions, the specific implementation adopted in the study may not fully capture the breadth and diversity of linear pedagogical frameworks.

Therefore, what is labeled as “linear pedagogy” in the present study appears to represent a simplified and restricted version of traditional instruction, rather than a comprehensive instantiation of linear pedagogical frameworks. This raises concerns about comparator validity, as the contrast may reflect differences in task design and implementation fidelity rather than differences between well-defined pedagogical traditions. This mismatch leads to a confounding comparison between a coherent, theoretically informed NLP condition and a narrow view of the LP condition. Consequently, the current comparison may contrast a highly representative and game-based practice condition with a more restricted and less representative instructional format, rather than testing distinct theoretical models. The central criterion for valid comparisons between pedagogical frameworks should not be theoretical allegiance but pedagogical fidelity—the degree to which an intervention authentically embodies the principles of its guiding theory. Future research should ensure that both interventions are equally aligned with their theoretical premises, allowing differences in learning outcomes to be meaningfully attributed to pedagogical principles rather than to task quality.

A potential way to address this issue is to incorporate measures of instructional and treatment validity throughout the intervention. For example, in a previous study comparing direct instruction with a hybrid TGfU–Sport Education approach, a 14-item observational scale was applied to a sample of sessions to assess the presence of key pedagogical principles (Gil-Arias et al., 2017). Similar procedures could be implemented to evaluate the quality and fidelity of interventions in both groups, allowing for better control of potential biases arising from theoretical misrepresentation.

## 2 Control of game exposure and task similarity

A central limitation concerns the lack of control over actual game time and the similarity between training and testing conditions. The authors report that the main performance test consisted of a 5-a-side match (40 min) used in both pre-, post-, and retention tests, which closely mirrors the practice structure of the NLP sessions (small-sided, representative tasks) but diverges substantially from the LP sessions, which predominantly involved isolated drills. Consequently, the testing environment may favor the NLP group due to the principle of specificity of learning, although the magnitude of this effect cannot be determined without quantifying task exposure and similarity across conditions. A previous literature review showed that SSG-based interventions showed better learning outcomes than control, non-game-based interventions (Clemente et al., 2021). This result has been replicated recently by other experiments (Deuker et al., 2024). Therefore, the between-group differences reported by Yang et al. (2025) may be attributed to the high similarity between the NLP training and the testing tasks rather than to the theoretical foundation of the intervention itself. Another control group, with equal playing time (but, for example, without the progression and interventions argued to be NLP-specific), would strengthen such conclusions. In this context, reporting the proportion of time spent in game-based activities, the number of player interactions, or the degree of structural similarity between training and testing tasks would allow a more precise assessment of the extent to which specificity may have influenced the results.

Prior research has emphasized the need to balance task representativeness across groups or include transfer tasks to ensure fair comparisons (Fleddermann et al., 2019; Deuker et al., 2024). Without quantifying or equating total game-play time, it is difficult to determine whether observed improvements reflect true pedagogical effects or simply contextual familiarity with the testing task.

## 3 Lack of control over effective practice time

A further methodological issue relates to unequal active engagement time. Although Supplementary Table 1 suggests differences in task organization, the absence of quantitative indicators such as time-on-task, number of repetitions, or player involvement ratios limits the ability to estimate the magnitude of these differences and their potential impact on performance outcomes. As presented in Supplementary Table 1, LP participants often practiced sequentially in lines (“two lines at the same time,” “one by one through cones”), while NLP sessions involved simultaneous play among several participants (e.g., 8–10 players interacting in constrained games). As a result, NLP players likely accumulated substantially greater effective practice time and interaction opportunities. Reporting the effective practice time would mitigate this problem and help assess comparability.

From a practical perspective, besides reporting effective practice time, researchers in intervention studies could improve reproducibility by adopting some methodological strategies. For example, recording task density or active engagement ratios (i.e., ball-contact or decision-making time per player) could facilitate the control of the influence of effective practice time on the reported variables. Differences in total practice exposure could partially explain the superior performance of the NLP group, independently of its pedagogical nature.

## 4 Implications for interpretation and external validity

Given these methodological confounds (unequal representativeness, exposure, and engagement), the conclusion that NLP is categorically superior to LP should be interpreted with caution. The observed advantages may stem from practice specificity and contextual similarity, rather than from the inherent superiority of the non-linear framework. To advance the scientific discussion and empirical evidence regarding NLP, future research should consider the following design refinements:

1. Match exposure control: Quantify and equate time spent in game-like activities and ensure equivalent opportunities for contextualized decision-making across conditions.
2. Task representativeness audit: Use independent expert panels to verify that each task maintains an appropriate degree of representativeness relative to the competition environment.
3. Effective engagement measurement: Record and report players' active practice time and the number of task repetitions per session. This would allow for a better comprehension of the intervention effects and facilitate replication (Afonso et al., 2026).

Importantly, the original study examined the moderating role of adaptability, which represents a meaningful contribution beyond a simple comparison between pedagogical approaches and contributes to our understanding of how individual adaptability interacts with instructional formats to shape learning outcomes. However, the methodological constraints discussed above may also influence the interpretation of this finding. For example, if the NLP condition provided greater exposure to variable and representative contexts, this may have disproportionately benefited participants with higher adaptability, not necessarily because of the pedagogical framework *per se*, but due to the interaction between individual characteristics and task design. Therefore, the reported moderation effect should be interpreted in light of potential differences in practice structure and exposure.

## 5 Conclusion

Yang et al.'s study offers valuable insights into the role of adaptability in motor skill learning and contributes to an important discussion within sport pedagogy. However, limitations related to task representativeness, comparator design, and control of practice exposure suggest that the findings should be interpreted with caution. Rather than indicating a general superiority of non-linear pedagogy, the results may reflect differences in task design, contextual similarity, and opportunities for engagement across conditions. Future studies adopting more balanced and clearly operationalized pedagogical contrasts will be essential to clarify the mechanisms underlying these effects. At the same time, it is important to acknowledge that more structured, teacher-directed instructional formats may remain appropriate and pedagogically justifiable in contexts requiring high levels of control, safety, or standardization.
